# Genetic diversity of influenza A viruses circulating in Bulgaria during the 2018–2019 winter season

**DOI:** 10.1099/jmm.0.001198

**Published:** 2020-05-27

**Authors:** Neli Korsun, Rodney Daniels, Svetla Angelova, Burcu Ermetal, Iliyana Grigorova, Silvia Voleva, Ivelina Trifonova, Anna Kurchatova, John McCauley

**Affiliations:** ^1^​ National Laboratory “Influenza and ARI”, Department of Virology, National Centre of Infectious and Parasitic Diseases, 44A Stoletov Blvd, 1233 Sofia, Bulgaria; ^2^​ WHO Collaborating Centre for Reference and Research on Influenza, Worldwide Influenza Centre, The Francis Crick Institute, 1 Midland Road, London NW1 1AT, UK; ^3^​ Department of Epidemiology, National Centre of Infectious and Parasitic Diseases, 26 Yanko Sakazov Blvd, 1504 Sofia, Bulgaria

**Keywords:** amino acid substitution, genetic characterisation, influenza virus

## Abstract

**Introduction:**

Influenza viruses evolve rapidly and change their antigenic characteristics, necessitating biannual updates of flu vaccines.

**Aim:**

The aim of this study was to characterize influenza viruses circulating in Bulgaria during the 2018/2019 season and to identify amino acid substitutions in them that might impact vaccine effectiveness.

**Methodology:**

Typing/subtyping of influenza viruses were performed using real-time Reverse Transcription-PCR (RT-PCR) and results of phylogenetic and amino acid sequence analyses of influenza strains are presented.

**Results:**

A(H1N1)pdm09 (66 %) predominated over A(H3N2) (34 %) viruses, with undetected circulation of B viruses in the 2018/2019 season. All A(H1N1)pdm09 viruses studied fell into the recently designated 6B.1A subclade with over 50 % falling in four subgroups: 6B.1A2, 6B.1A5, 6B.1A6 and 6B.1A7. Analysed A(H3N2) viruses belonged to subclades 3C.2a1b and 3C.2a2. Amino acid sequence analysis of 36 A(H1N1)pdm09 isolates revealed the presence of six–ten substitutions in haemagglutinin (HA), compared to the A/Michigan/45/2015 vaccine virus, three of which occurred in antigenic sites Sa and Cb, together with four–nine changes at positions in neuraminidase (NA), and a number of substitutions in internal proteins. HA1 D222N substitution, associated with increased virulence, was identified in two A(H1N1)pdm09 viruses. Despite the presence of several amino acid substitutions, A(H1N1)pdm09 viruses remained antigenically similar to the vaccine virus. The 28 A(H3N2) viruses characterized carried substitutions in HA, including some in antigenic sites A, B, C and E, in NA and internal protein sequences.

**Conclusion:**

The results of this study showed the genetic diversity of circulating influenza viruses and the need for continuous antigenic and molecular surveillance.

## Introduction

Influenza viruses cause highly contagious infection, that occurs as annual epidemics affecting approximately 5–15 % of the human population, resulting in 3–5 million cases of severe illness and 290 000–650 000 deaths globally [1]. Since 2009, two subtypes of influenza A viruses, A(H1N1)pdm09 and A(H3N2), together with two genetic lineages of B viruses, B/Victoria and B/Yamagata, have been responsible for seasonal epidemics. Influenza A viruses cause moderate to severe illnesses, affect all age groups, have a large animal reservoir and undergo faster evolution than type B viruses. Type B viruses usually cause milder illnesses, most commonly affecting children and, in contrast to type A viruses, have neither an animal reservoir nor pandemic potential.

The genomes of influenza A and B viruses consist of eight segments of single-stranded RNA with negative polarity, each of them encoding at least one protein with specific function(s). The surface glycoproteins – haemagglutinin (HA) and neuraminidase (NA) – elicit protective antibodies and are major components of influenza vaccines. Under host immune pressure, amino acid substitutions accumulate in these glycoproteins, leading to escape from natural or vaccine-induced immune responses and to a decrease in vaccine effectiveness. This phenomenon, known as antigenic drift, necessitates twice-yearly updating of influenza vaccine composition in order to correspond to circulating strains. Amino acid substitutions underlying antigenic drift are mainly located in the so-called antigenic (antibody-recognized) sites of HA. Five distinct antigenic sites have been identified in the HAs of both A(H1N1) (Ca1, Ca2, Cb, Sa, Sb) and A(H3N2) (A–E) viruses [2–4]. A relatively small number of amino acid substitutions in sites surrounding the receptor binding site (RBS) may result in altered antigenic phenotype [5–7], but other substitutions also have significant impact on antigenicity [8]. Notably, substitutions that create potential glycosylation sequons, NX S/T where X can be any amino acid but P, in HA and NA is another mechanism that enables the virus to evade the immune system. It is suggested that the added host-derived glycans alter antigenicity of HA by masking antigenic epitopes and preventing antibody recognition [9, 10].

Laboratories of the World Health Organization (WHO) Global Influenza Surveillance and Response System (GISRS) perform continuous global surveillance of circulating influenza viruses with tracking of their evolutionary dynamics in order to optimize the selection of vaccine viruses. Analysis of the viruses identified allows early detection of novel genetic variants with altered antigenicity, epidemic potential, increased virulence or reduced susceptibility to antivirals. Based on this global surveillance, WHO makes recommendations twice a year, for countries in the Northern and Southern Hemispheres, respectively, for the composition of influenza vaccines in the forthcoming influenza season. The main *aims* of this study were to investigate the circulation pattern of influenza viruses causing the 2018/2019 season in Bulgaria and to determine their genetic and phenotypic characteristics for comparison with those of the vaccine and other epidemic viruses.

## Methods

### Study population and specimen collection

From October 2018 to May 2019, patients presenting with acute respiratory infection (ARI) from different regions of the country were enrolled in the national influenza surveillance program. ARI was defined in compliance with the European Centre for Disease Prevention and Control (ECDC) with sudden onset of symptoms, at least one of the following four respiratory symptoms: cough, sore throat, shortness of breath, coryza and a clinician's judgement that illness was due to an infection [11]. The diagnosis of each patient was determined by their attending physician based on standard clinical criteria. Nasopharyngeal specimens from the enrolled patients were collected using polyester collection swabs that were preserved in virus transport medium (Deltalab, Spain) during the visit to the doctor or within the first 24 h of admission. Swabs were stored at 4 °C for up to 72 h before shipment to the National Laboratory ‘Influenza and ARI’ for influenza virus detection and characterization. The laboratory is recognized as a WHO National Influenza Centre. It is the only laboratory in the country that conducts research on influenza viruses and performs year-round diagnostic testing of samples from outpatients and hospitalized patients to monitor the circulation of seasonal influenza viruses. Specimens were processed immediately or stored at −80 °C before testing.

### Extraction of viral nucleic acids and real-time RT-PCR

Virus RNAs were extracted automatically from the respiratory specimens using a commercial ExiPrep Dx Viral DNA/RNA kit and ExiPrep 16DX equipment (BioNeer, Korea) in accordance with the manufacturer’s instructions. Detection and typing/subtyping of influenza viruses were carried out by a real-time RT-PCR method using a kit – SuperScript III Platinum One-Step Quantitative RT-PCR (qRT-PCR) System (Invitrogen, USA). All samples were first tested for the presence of influenza A and B viruses. Those that were positive for influenza A were subsequently screened for A(H1N1)pdm09 and A(H3N2). Primers, probes and positive controls were provided by the International Reagent Resource, USA. Amplification was performed with a Chromo 4 thermal cycler (Bio-Rad) in accordance with the protocol recommended by CDC, Atlanta, USA (reverse transcription at 50 °C for 30 min, Taq inhibitor inactivation at 95 °C for 2 min, followed by 45 cycles of denaturation at 95 °C for 15 s and annealing/amplification at 55 °C for 30 s). A Ct value <38 was regarded as positive.

### Virus isolation and antigenic characterization

Real Time RT-PCR positive clinical specimens with Ct values <28 were inoculated onto cultures of Madin–Darby Canine Kidney (MDCK) and MDCK-SIAT1 (a cell line expressing increased levels of α2,6-sialyltransferase cells [12]). Cultures were incubated at 35 °C in a 5 % CO_2_ atmosphere and observed daily for 7 days for evidence of cytopathology. The presence of virus in culture was confirmed by haemagglutination assay following standard protocols using a 1 % suspension of guinea pig red blood cells. Cell-culture isolates of viruses detected at different stages of the epidemics, in different regions of the country and in patients of different age groups were selected for antigenic and genetic characterization. Antigenic testing was performed by the haemagglutination inhibition (HI) assay, in accordance with the WHO Manual, using vaccine viruses/antigens and their corresponding antisera provided by the WHO Collaborating Centres (WHO-CCs) in London and Atlanta [13]. More detailed HI assays of representative Bulgarian influenza isolates with panels of reference viruses and antisera were performed at the WHO-CCs in London and Atlanta. Virus isolates were identified as antigenically related to the vaccine virus if they showed no more than fourfold reduced HI titre with antiserum raised against the vaccine virus, as compared to the homologous titre. A reduction of at least eightfold in the HI titres was considered a signal of antigenic difference.

### Genetic characterization

HA and NA gene sequences of influenza viruses detected in Bulgaria during the 2018/2019 season were determined at the WHO-CC London. Whole-genome sequencing was carried out at WHO-CC, Atlanta. Sequences have been deposited in the EpiFlu database of the Global Initiative on Sharing All Influenza Data (GISAID) [14]. For phylogenetic analyses, sequences of study viruses, reference viruses with known genetic group identities and viruses representing different countries of Europe during the 2018/2019 season, were retrieved from the EpiFlu database of GISAID (Table S1, available in the online version of this article). Multiple alignments for HA and NA sequences were carried out using the muscle algorithm and maximum-likelihood phylogenetic trees were constructed using RaxML v8.2X [15], followed by annotation with amino acid substitutions defining nodes and individual virus gene products (indicated in parenthesis after virus names) using treesub [16]. Trees were visualized using FigTree [17] and highlighted using Adobe Illustrator CC 2015.3 [18]. Phylogenies relate to nucleotide alignments of coding sequences for HA gene products, with signal-peptide encoding and stop codons removed to give mature HA glycoprotein amino acid numbering.

### Deduced amino acid sequence analysis and prediction of *N*-glycosylation motifs

The amino acid sequences were generated by translating nucleotide sequences with the standard genetic code using mega (version 6.06) software [19]. The deduced amino acid sequences of the study strains were compared to those of vaccine strains and other reference strains to identify amino acid substitutions. The amino acid identity was calculated using FluSurver [20]. Putative *N*-glycosylation motifs in the HA and NA were predicted using the NetNGlyc 1.0 web Server to identify sequence motifs N–X–S/T (sequon), where X can be any amino acid except proline. Only threshold values of >0.5 were considered as potential glycosylation sites. The occupancy of glycosylation sites in the HAs of some recent H1 and H3 viruses has been determined [21, 22].

### Antiviral susceptibility surveillance

Screening of A(H1N1)pdm09 viruses for the presence of point mutations encoding NA H275Y amino acid substitution, known to confer oseltamivir resistance, was carried out using a real-time RT-PCR assay that allowed discrimination of a single nucleotide difference between oseltamivir sensitive and resistant viruses. Two TaqMan probes differing in position 823 of the NA gene were used simultaneously: the first probe contained a cytosine at position 823 and was labelled with VIC (H275), while the second probe contained thymine in the same position and was labelled with FAM (275Y). Primer/probe sequences and protocol were kindly provided by Public Health England, London. Reference influenza viruses A/Denmark/524/2009 (sensitive, H275) and A/Denmark/528/2009 (resistant, 275Y), provided by WHO-CC, London, were used as positive controls. A phenotypic analysis (MUNANA test) of influenza virus susceptibility to neuraminidase inhibitors (oseltamivir and zanamivir) was performed at WHO-CC, London.

### Statistics

Age and gender of patients, the clinical features of their illness and the incidence of each virus were compared using the Chi square or Fisher’s exact tests for categorical variables. *P-*values of <0.05 were considered statistically significant.

## Results

The 2018/2019 influenza epidemic lasted for 7 weeks (weeks 2–9/2019) and peaked during week 4/2019. The ARI incidence rate during the epidemic peak (247.92 per 10 000 population) was higher than the incidence rates during the epidemic peaks in the 2017/2018, 2016/2017 and 2015/2016 seasons (230.83, 215.52 and 158.74 per 10 000 population, respectively).

### Influenza virus detection

**Fig. 1.**
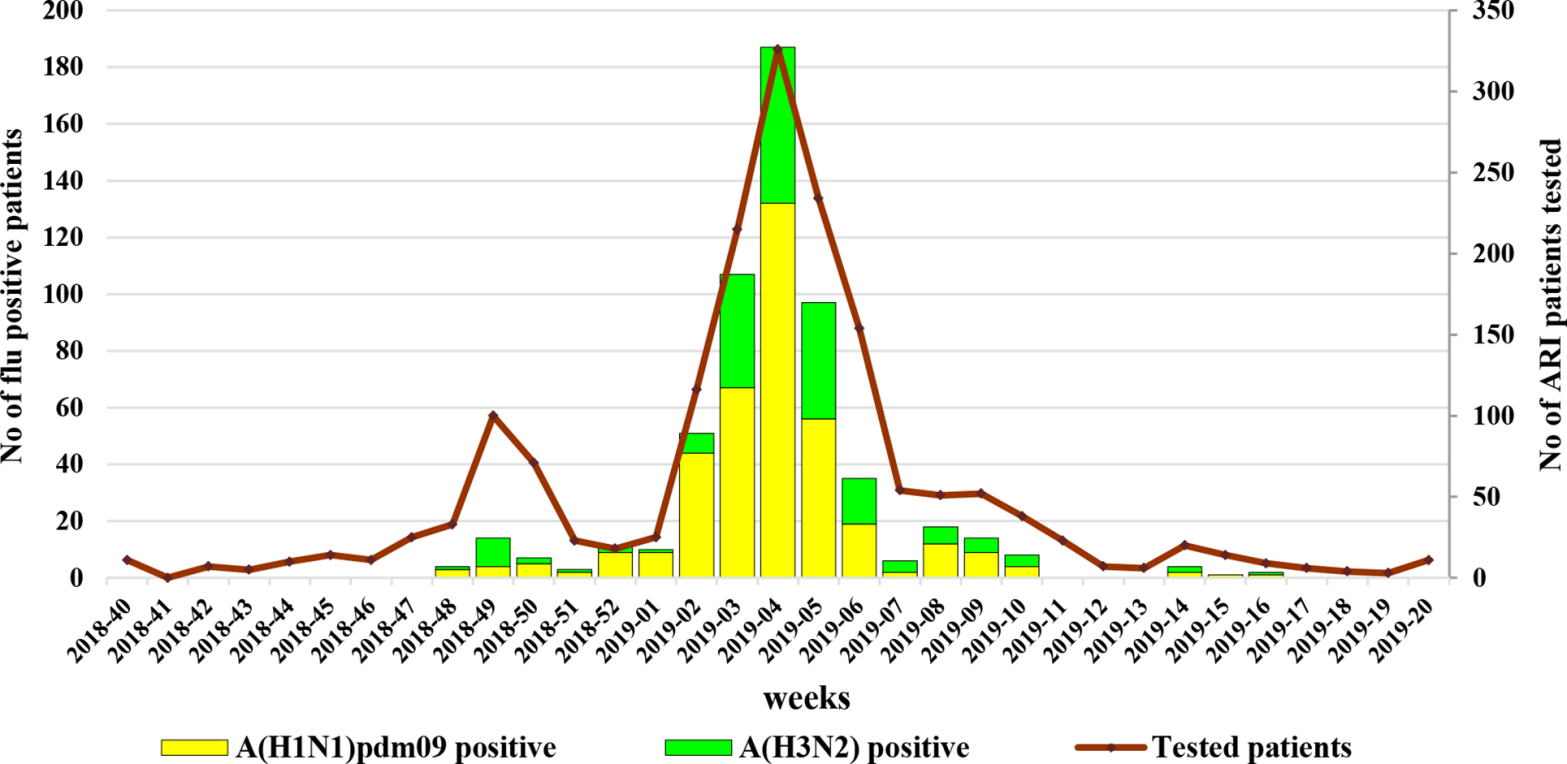
Weekly detections of influenza viruses in Bulgaria.

The study population consisted of 1696 patients presenting with ARI symptoms: 392 (23.1 %) of these were persons attending outpatient healthcare centrers; 1304 (76.9 %) were hospitalized patients, of which 71 were treated in intensive care units (ICU). The patients’ ages ranged from 14 days to 89 years (average age 28.8 years, median age 18 years) and 49.9 % were male. Influenza viruses were detected in samples from 578 (34.1 %) individuals of the study population with rates of infection being similar in outpatients (125/392; 31.9 %) and hospitalized patients (453/1304; 34.7 %). Of these, 380 (65.7 %) were positive for influenza A(H1N1)pdm09 and 198 (34.3 %) for A(H3N2) viruses. Infection rates for the two virus subtypes in the outpatient and hospitalized patient groups were as follows: for A(H1N1)pdm09 18.9 % (74/392) and 23.5 % (306/1304) (*P*=0.06) and for A(H3N2) 13 % (51/392) and 11.3 % (147/1304), respectively. In an immunocompromised patient, a mixture of influenza A(H1N1)pdm09 and A(H3N2) was found. No type B viruses were identified. Between weeks 50/2018 and 4/2019 and in weeks 8–9/2019, A(H1N1)pdm09 viruses dominated representing up to 90 % of the detected influenza viruses. The first influenza detection, an A(H1N1)pdm09 virus, occurred in week 48/2018, and the last, an A(H3N2) virus, in week 16/2019 (Fig. 1).

### Demographics and clinical characteristics of patients infected with influenza viruses

**Fig. 2.**
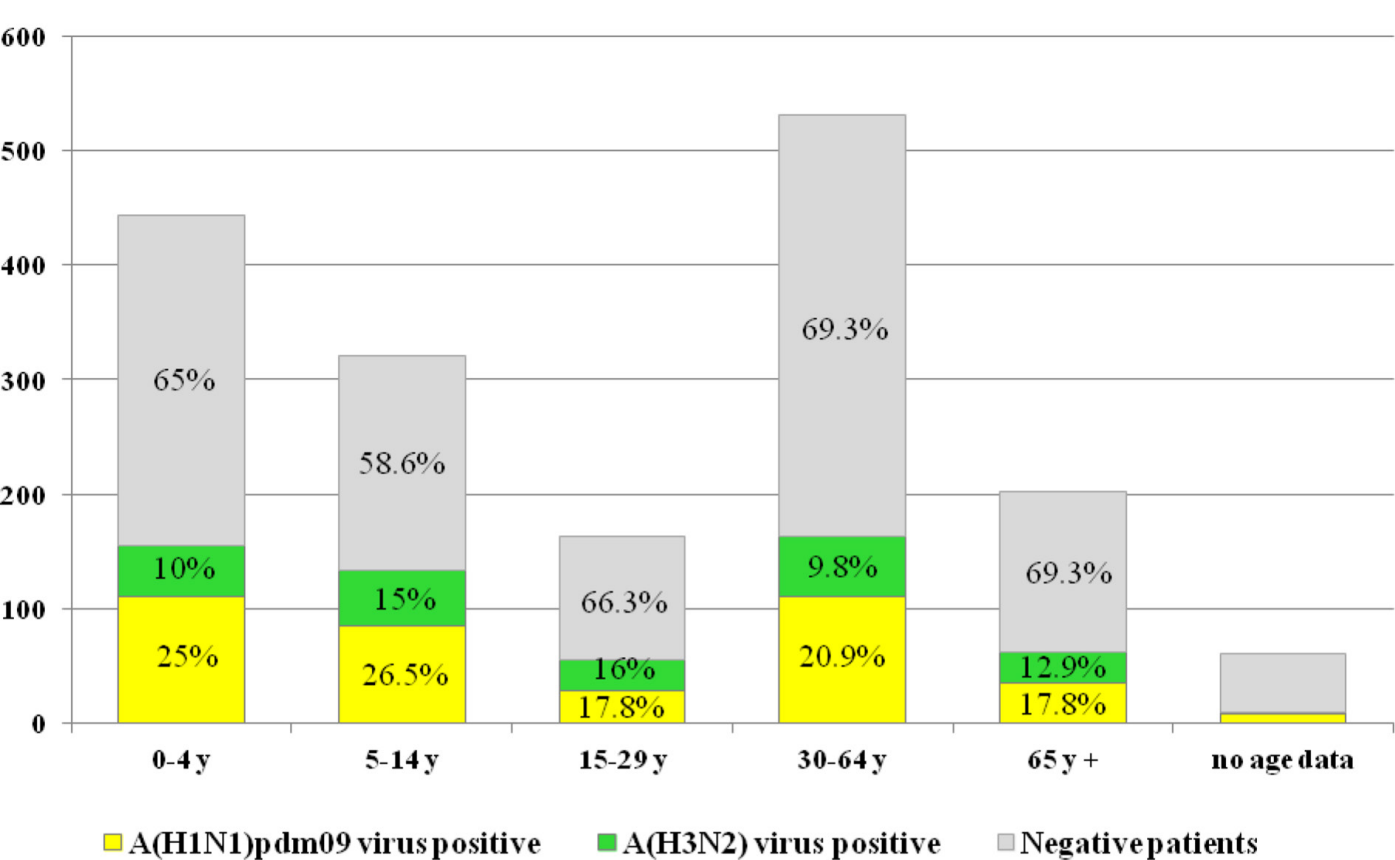
Age distribution of patients tested for influenza virus. Percentages of influenza-positive and -negative patients in each age group were calculated based on the total number of patients in the same age group.

All age groups were affected by influenza infection. The average age of influenza virus-positive patients was 26.74 years (range, 2 months to 89 years) and 52.4 % were male. The highest influenza virus positivity (41.7 %) was found in the patients aged 5–14 years and the lowest (30.7 %) in two adult groups aged 30–64 years and ≥65 years. The mean ages of patients infected with A(H1N1)pdm09 and A(H3N2) viruses were 25±24.73 years and 28.47±26.65 years, respectively; the median ages were 12 years and 16.5 years, respectively. A(H1N1)pdm09 viruses predominated and were most frequently detected in the 5–14 year (26.6 %) and 0–4 year (25.1 %) age groups. The proportions of A(H1N1)pdm09 viruses detected in patients aged 15–29 years and ≥65 years were equal (17.8 %). The highest rates of influenza A(H3N2) detection were in the 15–29 year (16 %) and 5–14 year (15 %) age groups (Fig. 2).

Antiviral drug (oseltamivir) was administered to all patients on admission to hospital and 67 % of outpatients received such a prescription.

Influenza viruses were detected in 28.8 % (68/236) of the studied patients diagnosed with pneumonia and in 23.6 % (33/140) of patients with CNS involvement (meningitis, encephalitis, brain edema, encephalopathy). Among infected patients with pneumonia, A(H1N1)pdm09 and A(H3N2) positive cases accounted for 76.5 % (52/68) and 23.5 % (16/68), respectively; and among infected patients with neurological complications, 51.5 % (17/33) and 48.5 % (16/33), respectively. Among the 71 patients treated in ICUs, A(H1N1)pdm09 and A(H3N2) viruses were detected in 21.1 % (15/71) and 2.8 % (2/71), respectively. Of the 17 studied patients with fatal outcomes of infection, 10 and 2 were infected with A(H1N1)pdm09 and A(H3N2) viruses, respectively. Among the patients with confirmed influenza infection who were treated in ICUs or who died, 3/17 and 3/12, respectively were ≥65 years of age, with the remainder were aged 30–64; all were unvaccinated.

More hospitalized patients, patients treated in ICUs and deceased patients were tested for influenza in the 2018/2019 than in the previous 2017/2018, 2016/2017 and 2015/2016 seasons with positive results for influenza viruses as follows: 453/1304 (34.7 %), 341/1070 (31.9 %), 185/665 (27.8 %) and 279/909 (30.7 %) for hospitalized patients; 17/71, 6/31, 6/31 and 13/36 for patients treated in ICUs; and 12/17, 3/6, 0/1 and 2/8 for deceased patients.

### Genetic and antigenic characterization

**Fig. 3.**
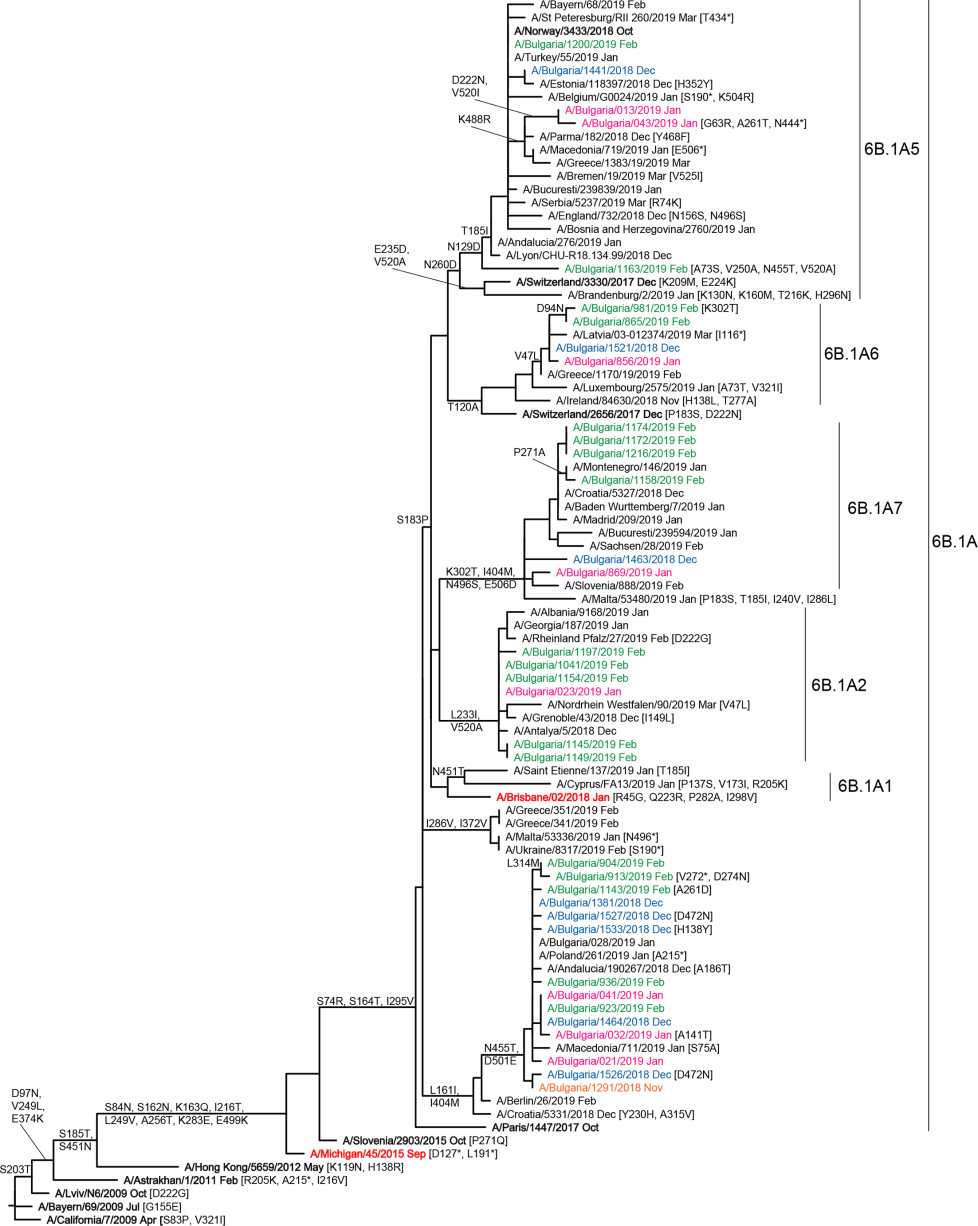
HA gene phylogeny of influenza A(H1N1)pdm09 viruses detected in Bulgaria during the 2018/2019 season. The tree is rooted at A/California/7/2009. Reference viruses are indicated in bold and vaccine virus A/Michigan/45/2015 in red. Bulgarian viruses detected from November 2018 to February 2019 are indicated in orange, blue, pink and green, respectively. All but one virus (A/Bulgaria/1163/2018) viruses were detected in hospitalized patients. Two viruses: A/Bulgaria/1463/2018 and A/Bulgaria/869/2019 (both 6B.1A7) were recovered from patients who died. All viruses were detected in unvaccinated patients.

A total of 69 influenza viruses (5 in clinical specimens and 64 as cell-culture isolates) were sequenced at WHO-CCs in London and Atlanta and deposited in the EpiFlu database of GISAID. Phylogenetic trees were constructed to determine the genetic relationships of Bulgarian viruses with vaccine viruses and viruses circulating in other countries in the same period. Phylogenetic analysis showed that HA and NA genes of all 36 sequenced Bulgarian A(H1N1)pdm09 viruses belonged to subclade 6B.1A, which emerged in the northern hemisphere winter of 2016/2017, with a number falling in four different genetic groups within subclade 6B.1A: 6B.1A5 (five viruses), 6B.1A6 (four viruses), 6B.1A7 (six viruses) and 6B.1A2 (six viruses) (Fig. 3). All Bulgarian A(H1N1)pdm09 viruses were antigenically similar to the vaccine virus, A/Michigan/45/2015, as defined by post-infection ferret antisera in HI assays.

**Fig. 4.**
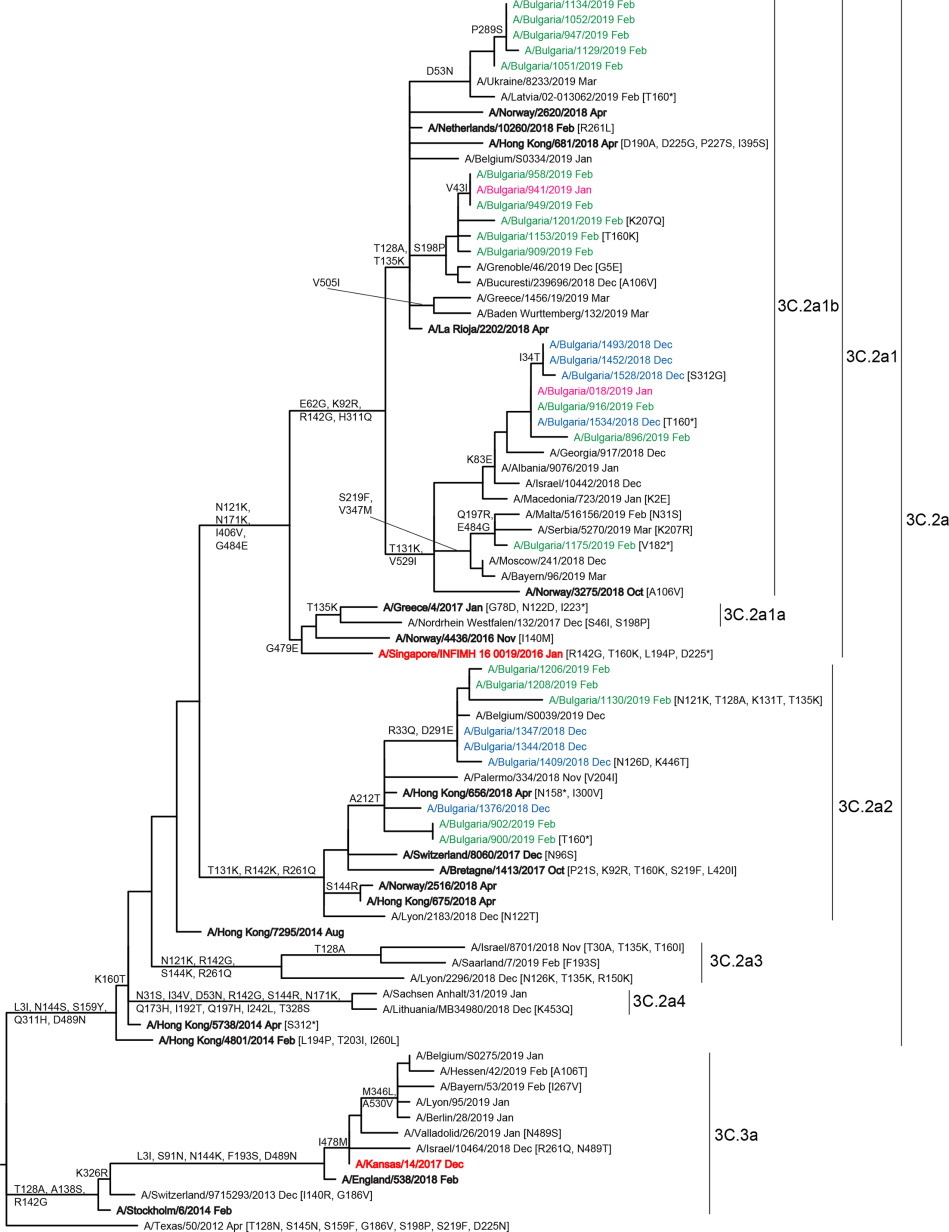
HA gene phylogeny of influenza A(H3N2) viruses detected in Bulgaria during the 2018/2019 season. The tree is rooted at A/Texas/50/2012. Reference viruses are indicated in bold and vaccine viruses A/Singapore/INFIMH-16-019/2016 and A/Kansas/14/2017 in red. Bulgarian viruses detected from December 2018 to February 2019 are indicated in blue, pink and green, respectively. All but six viruses: A/Bulgaria/1344/2018, A/Bulgaria/1347/2018, A/Bulgaria/1376/2018 and A/Bulgaria/1409/2018 (all subclade 3C.2a2), A/Bulgaria/909/2019 and A/958/2019 (both subgroup 3C.2a1b) viruses were detected in hospitalized patients. All viruses were identified in unvaccinated patients.

All 28 genetically characterized A(H3N2) viruses fell into genetic clade 3C.2a. Within this clade, 19 viruses belonged to the A/Alsace/1746/2018 (3C.2a1b) subgroup and 9 belonged to the A/Switzerland/8060/2017 (3C.2a2) subclade (Fig. 4). Antigenic characterization of clade 3C.2a viruses using HI assay was difficult because the majority of these viruses did not agglutinate red blood cells. Ten Bulgarian subgroup 3C.2a1b and two subclade 3C.2a2 viruses analysed by HI with post-infection ferret antisera showed low reactivity with antisera raised against the egg-propagated vaccine virus A/Singapore/INFIMH-16-019/2016 (subclade 3C.2a1).

### Amino acid sequence comparisons

Sequences of full-length HA and NA glycoproteins of Bulgarian influenza A(H1N1)pdm09 and A(H3N2) viruses (36 and 28, respectively) were compared to those of egg-propagated vaccine viruses to identify substitutions that might impact vaccine effectiveness.

*A(H1N1)pdm09*. HA amino acid sequence identity of studied isolates ranged from 97.3 to 98.4 % compared to the vaccine virus, A/Michigan/45/2015. All 36 A(H1N1)pdm09 HA sequences contained three amino acid changes in HA1, S74R, S164T and I295V, that were fixed in globally circulating subclade 6B.1A viruses (Table 1) [23].

**Table 1.** Amino acid substitutions in HA and NA of influenza viruses, detected in Bulgaria during the 2018/2019 season

Amino acid changes in HA and NA														
**A(H1N1)pdm09 sequences (*n*=36) compared to A/Michigan/45/2015**														
HA	6B.1A5 (5)	**S74R** (5) *Cb*	**S164T** (5) *Sa*	**I295V** **(5)**	N129D (5) ***Sa***	S183P (5)	T185I (4)	D222N (2)	N260D (5)	*K161R* (2)	*V193I* (2)	**R223Q** **(5)**		
	6B.1A6 (4)	**S74R** (4) *Cb*	**S164T** (4) *Sa*	**I295V** **(4)**	V47L (4)	D94N (2)	T120A (4)	S183P (4)				**R223Q** **(4)**		
	6B.1A7 (6)	**S74R** (6) *Cb*	**S164T** (6) *Sa*	**I295V** **(6)**	S183P (6)	K302T (6)	*I77M* (6)	*N169S* (6)	*E179D* (6)			**R223Q** **(6)**		
	6B.1A2 (6)	**S74R** (6) *Cb*	**S164T** (6) *Sa*	**I295V** **(6)**	S183P (6)	L233I (6)	*V193A* (6)					**R223Q** **(6)**		
	6B.1A - Outlier (15)	**S74R** (15) *Cb*	**S164T** (15) *Sa*	**I295V (15)**	L161I (15)	L314M (2)	*I77M* (15)	*N128T* *(15)*	*D145N* (2)+CHO	*D174E* (15)		**R223Q** **(15)**		
na	6B.1A5 (5)	**G77R** **(5)**	**V81A** **(5)**	**N449D** **(5)**	Q51K (5)	V67I (3)	F74S (5)	I188T (5)	D416N (3)	D416K (2)	T452I (5)			
	6B.1A6 (4)	**G77R** **(4)**	**V81A** **(4)**	**N449D** **(1)**	T72I (4)	S79P (4)	I188T (3)	I365T (4)	D416N (4)					
	6B.1A7 (5)	**G77R** **(5)**	**V81A** **(5)**	**N449D** **(5)**	M314I (5)									
	6B.1A2 (6)	**G77R** **(6)**	**V81A** **(6)**	**N449D (6)**	F74L (6)	L85I (6)	I188T (6)	M314I (2)	I436V (6)					
	6B.1A- Outlier (15)	**G77R (15)**	**V81A (15)**	**N449D** **(15)**	F74L (15)	S105N (15)	I188T (15)	I436V (14)						
**A(H3N2) sequences (*n*=28) compared to A/Singapore/INFIMH-16-0019/2016**														
HA	3C.2a1b +T135K (11)	V43I (3)	D53N (5) ***C***	E62G (11) ***E***	K92R (11)	T128A(11) -CHO ***A***	T135K (11) –CHO ***A***	S198P (6)	P289S (4)	H311Q (11)	*E150G* (11)	**K160T (10)** **+CHO *B***	**P194L** **(11)**	**G225D (11)**
	3C.2a1b +T131K (8)	I34T (3)	E62G (8) ***E***	K83E (7) ***E***	K92R (8)	T131K (8)	H311Q (8)	*E150G* (8)	*V200I* (8)			**K160T (8)** **+CHO *B***	**P194L** **(8)**	**G225D (8)**
	3C.2a2 (9)	R33Q (6)	K121N (8)	T131K (8)	G142R (9) ***A***	K171N (9)	A212T (9)	R261Q (9)	D291E (6)	*V77I* (9)	*E150G* (9)	**K160T (9)** **+CHO *B***	**P194L** **(9)**	**G225D (9)**
na	3C.2a1b +T135K (10)	G93S (5)	P126L (10)	I212V (10)	K220N (10)	N234D (5)-CHO	V263I (5)	V303I (10)	N329S (10)-CHO	L338F (6)	V398I (5)	D399N (5)		
	3C.2a1b +T131K (7)	P126L (7)	I212V (7)	K220N (7)	V303I (7)	S315R (7)	N329S (7)-CHO	E344K (7)	G346D (7)					
	3C.2a2 (9)	T72N (2)-CHO	I176M (9)	I212V (9)	H264Y (2)	N329S (9)-CHO	N339K (2)	P386S (9)						

Only amino acid substitutions found in two or more viruses are presented in this table. The number of Bulgarian influenza sequences possessing the substitution is indicated within parentheses. The amino acid substitutions defining the subclade/subgroup are shown in grey shading. For A(H1N1)pdm09, amino acid substitutions that define HA and NA of subclade 6B.1A viruses are shown in bold type. Substitutions in HA2 are identified in italics using HA2 numbering used; substitutions in antigenic sites are identified in bold italic and red colour after parentheses. Gain/loss of an *N*-glycosylation motif is indicated by +/-CHO. On the right of the table the reversal of amino acid substitutions associated with egg-adaptation of vaccine viruses are indicated in bold type; no such substitutions were observed in NA. Sequences contained in GISAID isolate identifiers EPI_ISL_237793 (A/Michigan/45/2015) and EPI_ISL_344420 (A/Singapore/INFIMH-16-0019/2016) were used to make the comparisons.

All studied viruses that fell in subgroups of subclade 6B.1A carried HA1 S183P substitution. Bulgarian representatives of the individual subgroups and ‘outlier’ group possessed different amino acid substitutions defining these subgroups (Table 1) [23]. Of the amino acid substitutions, three were located in antigenic sites: S74R in site Cb; S164T and N129D in site Sa [24]. Nine conserved potential *N*-glycosylation motifs were encoded by the HA genes of the 36 Bulgarian A(H1N1)pdm09 viruses: at HA1 positions 10/11, 23, 87, 162, 276, 287 and HA2 positions 154 and 213. The substitution S164T modified the 162–164 sequon, NQS→NQT, within the Sa antigenic site.

NA sequences available for 35 Bulgarian A(H1N1)pdm09 viruses differed from that of A/Michigan/45/2015 at several amino acid positions. Common substitutions in viruses of all genetic subgroups were G77R, V81A and N449D. Subgroup defining combinations of substitutions are presented in Table 2. The substitutions L85I, S105N, I188T, N449D and T452I occur at amino acid positions associated with NA antigenic sites [25, 26]. NA catalytic site residues (R118, D151, R152, R224, E276, R292, R371, Y406) and framework residues, which support the catalytic site (E119, R156, W178, S179, D/N198, I222, E227, H274, E277, N294) were conserved in all Bulgarian A(H1N1)pdm09 viruses [27], as were eight potential *N*-glycosylation motifs at positions 42, 50, 58, 63, 68, 88, 146 and 235.

**Table 2.** Amino acid substitutions in the internal proteins of A(H1N1)pdm09 viruses

Internal proteins	Amino acid substitutions	Bulgarian strains carrying these substitutions	No. of strains detected in other Balkan countries carrying these substitutions
PB2	T81I; G225S; V667I	A/Bulgaria/013/2019	Albania (4), Macedonia (7)
	R299K; T398I; P453T	All 5 Bulgarian strains	Albania, Macedonia, Serbia (All)
PB1	V200I; K386R	A/Bulgaria/013/2019	Albania (4), Macedonia (7)
PA	C8S; R356K; D394N; E688G	A/Bulgaria/1381/2018; A/Bulgaria/1527/2018	
NP	V319I	A/Bulgaria1381/2018; A/Bulgaria/1527/2018	Albania (1), Macedonia (3)
	V431I	A/Bulgaria/013/2019	Albania (4), Macedonia (7)
M1	A166T	A/Bulgaria1381/2018; A/Bulgaria/1527/2018	
M2	E14G	A/Bulgaria/1463/2019	Serbia (3)
	S23N	A/Bulgaria/013/2019	Albania (4), Macedonia (7)
NS1	M65T	All 5 Bulgarian strains	Albania, Macedonia, Serbia (All)
	T80A	A/Bulgaria/013/2019	Albania (4), Macedonia (7)
	I111M	A/Bulgaria/1463/2019	Serbia (3)
	A155T	A/Bulgaria/013/2019	Albania (3), Macedonia (7)

Five Bulgarian viruses (A/Bulgaria/1381/2018, A/Bulgaria/1521/2018, A/Bulgaria/1527/2018, A/Bulgaria/013/2019 and A/Bulgaria/021/2019) underwent whole-genome sequencing. Encoded internal protein sequences were compared with those of A/Michigan/45/2015 (EPI_ISL_200780) and other viruses from Albania (EPI_ISL_380103 - EPI_ISL_380107), Macedonia (EPI_ISL_362268 - EPI_ISL362274) and Serbia (EPI_ISL_393798 - EPI_ISL_393802).

Protein sequences derived from the other six genes (internal proteins) of five Bulgarian A(H1N1)pdm09 viruses were compared to those of A/Michigan/45/2015 vaccine virus and A(H1N1)pdm09 viruses detected in Albania, Macedonia and Serbia, where severe and fatal influenza cases were also reported during the 2018/2019 epidemic (Table 2). Bulgarian isolates contained amino acid substitutions in all internal proteins, most being in PB2. Some of these substitutions were detected in other Balkan countries but the possible functional importance of these substitutions is not known. As with the vast majority of A(H1N1)pdm09 viruses, M2 proteins carried S31N substitution associated with resistance to M2-ion channel blockers (amantadine and rimantadine) [28].

*A(H3N2).* Complete HA protein sequences of 28 Bulgarian viruses were analysed and compared with the northern hemisphere 2018/2019 vaccine virus A/Singapore/INFIMH-16-0019/2016 (subclade 3C.2a1). The similarity of Bulgarian viruses with the vaccine virus at the HA amino acid level ranged from 97.1 to 98.2 %. All 28 Bulgarian viruses carried HA2 E150G substitution, the 19 subclade 3C.2a1b viruses were characterized by three HA1 substitutions, and the nine subclade 3C.2a2 viruses by four HA1 and one HA2 substitutions (Table 1). The subclade 3C.2a1b viruses can be subdivided into two genetic groups: 11 with HA1 T128A and T135K substitutions and eight with HA1 T131K and HA2 V200I substitutions. Several substitutions were located in antigenic sites: three in site A; one in site C; two in site E. Of the three egg-adaptation substitutions in the A/Singapore/INFIMH-16-0019/2016 vaccine virus (T160K resulting in the loss of a glycosylation sequon in antigenic site B, L194P in the 190-helix (residues 188–194) and D225G within the RBS), one Bulgarian virus (A/Bulgaria/1153/2019) carried T160K substitution and two (A/Bulgaria/1534/2018 and A/Bulgaria/900/2019) showed polymorphism at this position (T160X). Thirteen potential *N*-glycosylation motifs in HA (HA1 positions 8, 22, 38, 45, 63, 122, 126, 133, 158, 165, 246 and 285, and HA2 position 154) were identified. Substitutions T160K/X (in antigenic site B), T128A and T135K (in antigenic site A) result in loss of *N*-glycosylation sequons, HA2 E155G falls within an *N*-glycosylation sequon (NET→ NGT).

NAs of Bulgarian A(H3N2) viruses all carried I212V and N329S substitutions compared to A/Singapore/INFIMH-16-019/2016. The 17 HA subclade 3C.2a1b viruses and the nine HA subclade 3C.2a2 viruses possessed different amino acid substitutions defining these subgroups (Table 1). The catalytic site and supporting framework residues within NA were highly conserved, as were seven of eight potential *N*-glycosylation sequons (at positions 61, 70, 86, 146, 200, 234, 245 and 367), two of which (146 and 367) locate close to the enzyme active site [29]. Substitutions T72N, N234D and N329S identified in some or all Bulgarian viruses resulted in the loss of *N*-glycosylation sequons.

Internal proteins sequences of four Bulgarian A(H3N2) viruses were compared to those of A/Singapore/INFIMH-16-0019/2016 vaccine virus and A(H3N2) viruses detected in Albania (5) and Macedonia (9). Amino acid substitutions were detected at six PB2, three PA, two NP, one each PB1, M2 and NS1 amino acid positions.

### Antiviral susceptibility surveillance

Of the Bulgarian viruses assessed for susceptibility to NA (oseltamivir, zanamivir) and PA (baloxavir marboxil) inhibitors, none showed evidence of reduced susceptibility. Testing was performed on 280 detected A(H1N1)pdm09 viruses by real-time RT-PCR with respect to the NA H275Y oseltamivir resistance substitution at the NRL in Bulgaria; NA and PA sequences of all study A(H1N1)pdm09 and A(H3N2) viruses were screened for known markers of reduced susceptibility to NA inhibitors and baloxavir marboxil [30, 31] and phenotypic testing (by the MUNANA method) was performed on 13 influenza A(H1N1)pdm09 and 5 A(H3N2) influenza viruses.

## Discussion

The 2018/2019 influenza epidemic in Bulgaria was characterized by moderate intensity but more specimens from severe and fatal influenza cases were examined compared to the previous three seasons. According to media reports, greater numbers of influenza deaths were also reported in other Balkan Peninsula countries (Romania, Croatia, Serbia and others) during the same period. The total number and percentage of influenza positive cases were slightly higher (34 vs 33 %) compared to the previous 2017/2018 season, however, among the hospitalized patients, the difference was greater (34.7 vs 31.9 %). Only influenza A(H1N1)pdm09 and A(H3N2) viruses, at a ratio of 2 : 1, were identified in contrast to the previous season when type B viruses (mainly B/Yamagata-lineage) predominated accounting for 80 % of all influenza cases [32]. A similar proportion amongst circulating influenza viruses was observed in most European countries. Cumulative data for the WHO European region showed strong prevalence of influenza A viruses in the 2018/2019 influenza season accounting for 99 % of all positive influenza cases with A(H1N1)pdm09 prevailing over A(H3N2) [33].

Generally, influenza A viruses account for the majority of influenza cases, prevail in most seasons, cause a higher percentage of serious illness and more frequently undergo genetic and antigenic changes in comparison to influenza B viruses [7]. In Bulgaria, influenza A was dominant in all but two (2012/2013 and 2017/2018) seasons following the 2009/2010 pandemic. Clear-cut dominance of influenza A(H1N1)pdm09 viruses was observed during the 2010/2011, 2013/2014 and 2015/2016 epidemics, whilst A(H3N2) viruses prevailed strongly in 2011/2012, 2014/2015 and 2016/2017 [34]. During the 2018/2019 epidemic, influenza A(H1N1)pdm09 viruses predominated, were most prevalent among children aged 5–14 years and were responsible for a higher proportion of serious illnesses than A(H3N2) viruses, which most severely affected 15–29 year (16%) and 5–14 year (15%) age groups. Analysis revealed increased proportions of influenza A(H1N1)pdm09 virus infection among hospitalized patients, among those treated in ICUs and among fatal influenza cases compared to proportions related to A(H3N2) infection.

Since the emergence of influenza A(H1N1)pdm09 virus during the 2009/2010 pandemic, its HA gene has undergone significant genetic changes and evolved in eight genetic groups and several clades/subclades. The recent clade 6B.1 viruses emerged during the 2015/2016 season and caused many severe and fatal influenza cases in a number of countries, including Bulgaria [35–37]. The most recently circulating viruses formed a subclade designated 6B.1A and seven subgroups have been defined within it: 6B.1A1 - 6B.1A7. A(H1N1)pdm09 viruses circulating in Bulgaria in 2018/2019, in contrast to previous seasons, were genetically diverse falling in four of these subgroups and a 6B.1A outlier group carrying several HA and NA amino acid substitutions. A total of 24 different substitutions (three located in antigenic sites Sa and Cb and one associated with egg-adaptation of the vaccine virus) were identified in HA and 18 in NA compared to vaccine virus, A/Michigan/45/2015. One to two amino acid changes in HA antigenic site Sa dramatically affect antibody recognition [38]. Gao *et al.* showed that N449D is one of three NA substitutions resulting in loss of reactivity with some human monoclonal antibodies [39]. Subgroup 6B.1A5 viruses prevailed globally and carried more substitutions in antigenic sites than other subgroups. Despite these changes in functionally important sites of HA and NA, subclade 6B.1A A(H1N1)pdm09 viruses remained antigenically similar to vaccine virus as assessed by use of post-infection ferret antisera raised against the A/Michigan/45/2016 vaccine virus [23]. However, recent subclade 6B.1A A(H1N1)pdm09 viruses with HA S183P substitution [58 % of the sequenced Bulgarian A(H1N1)pdm09 viruses] were antigenically distinguishable from the vaccine virus by panels of post-vaccination human sera, and this resulted in the choice of A/Brisbane/02/2018 as a vaccine component for the Northern Hemisphere 2019/2020 influenza season [40].

A(H1N1)pdm09 HA1 D222G/N/S or Q293H substitutions have been associated with cases of severe disease and fatalities earlier in the pandemic period [41]. D222G/N polymorphism has been reported in recently circulating viruses [42, 43] and two of the recent Bulgarian viruses (A/Bulgaria/013/2019 and A/Bulgaria/043/2019) carried D222N substitution. The D222G substitution was shown to cause a shift from α2,6-sialic acid receptor specificity to mixed α2,3/α2,6-sialic acid receptor specificity, adduced thereby to facilitate lung infection [44, 45].

Among the seasonal influenza viruses, A(H3N2) undergo the fastest evolution and diversification [7, 46]. Since the 2013/2014 Northern Hemisphere influenza season two genetic clades, 3C.2a and 3C.3a, have been in circulation. The majority of clade 3C.2a viruses showed poor or total loss of ability to agglutinate red blood cells, so it was difficult or impossible to assess the correlation between amino acid/glycosylation changes and the antigenic properties of these viruses using HI assay resulting in the implementation of virus neutralization assays [23]. During the recent influenza epidemics, several subclades (3C.2a1, 3C.2a2, 3C.2a3 and 3C.2a4) and subgroups (3C.2a1b and 3C.2a1a) have been identified within clade 3C.2a with subgroup 3C.2a1b viruses being predominant in the 2018/2019 season [47]. The majority (67.9 %) of Bulgarian A(H3N2) isolates belonged to subgroup 3C.2a1b, the remainder (32.1%) to subclade 3C.2a2. Bulgarian subgroup 3C.2a1b viruses showed amino acid substitutions at 17 positions in HA (inclusive of three associated with egg-adaptation) and 15 in NA, compared to the 2018/2019 vaccine virus, while subclade 3C.2a2 viruses were genetically closer to the vaccine virus with substitutions at 13 HA (inclusive of three associated with egg-adaptation) and 7 NA positions. Of the A(H3N2) HA amino acid substitutions seven at positions in HA1 were located within antigenic sites A, B (T160K substitution associated with egg-adaptation), C and E, and these changes could potentially alter the antigenicity of the HA. Antigenic sites B and A, localized on the top of HA around the RBS are main targets of human neutralizing antibodies and single substitutions, which occurred at seven positions in these sites (145 in site A and 155, 156, 158, 159, 189, 193 in site B) have been responsible for major antigenic cluster transitions from 1968 to 2003 [5]. In our study no substitutions were found in any of these seven key positions. Attachment and loss of oligosaccharides in HA and NA can also alter the biological properties of the viruses and the glycosylation in the antigenic regions of HA has been reported to associate with increased virulence [48]. For example, the *N*-glycan at HA1 position 158 of recent A(H3N2) viruses is in the vicinity of the residues at positions 156, 158 and 159 that are responsible for major antigenic changes. Non-HA substitutions, e.g. V263I in NA and K196E in NS1, associated with increased clinical severity in some reports [49], were not identified in Bulgarian isolates. Overall, our studies show increased amino acid substitution in antigenically important sites and higher levels of glycosylation in H3-HA compared to H1-HA (Table 3) supporting other authors' observations that H3 viruses evolve more rapidly and display more diversity compared to H1 viruses, which necessitates more frequent updates for this component of influenza vaccines [7, 50, 51].

**Table 3.** Number of positions with amino acid changes compared to vaccine viruses and number of potential *N*-glycosylation motifs in HA and NA of influenza viruses circulating in Bulgaria during the 2018/2019 season

Influenza viruses	No. of positions with amino acid changes compared to vaccine viruses			*N*-glycosylation motifs	
	HA		NA	HA	NA
	Total no.	Positions in antigenic regions			
A(H1N1)pdm09	6–10	2	4–10	9	8
A(H3N2)	8–10	6	7–11	13	8

Internal proteins sequences of four Bulgarian A(H3N2) viruses were compared to those of A/Singapore/INFIMH-16-0019/2016 vaccine virus and A(H3N2) viruses detected in Albania (5) and Macedonia (9) (Table 4). Amino acid substitutions were detected at six PB2, three PA, two NP, one each PB1, M2 and NS1 amino acid positions.

**Table 4.** Amino acid substitutions in the internal proteins of A(H3N2) viruses

Internal proteins	Amino acid substitutions	Bulgarian strains carrying these substitutions	No. of strains detected in other Balkan countries carrying these substitutions
PB2	T64I	A/Bulgaria/1347/2018; A/Bulgaria/1376/2018;	Macedonia (1)
	I96V	All 4 Bulgarian strains	Albania, Macedonia (All)
	S107D, K299R, K340R, M410V	A/Bulgaria/1452/2018; A/Bulgaria/1534/2018	Albania (1), Macedonia (1–4)
PB1	E618D	A/Bulgaria/1452/2018; A/Bulgaria/1534/2018	Albania (1), Macedonia (1)
PA	L105F, V668I	All 4 Bulgarian strains	Albania, Macedonia (All)
	K158R	A/Bulgaria/1452/2018; A/Bulgaria/1534/2018	Albania (1), Macedonia (1)
NP	V197I	All 4 Bulgarian strains	Albania (1), Macedonia (6)
	L418I	A/Bulgaria/1452/2018; A/Bulgaria/1534/2018	Albania (1), Macedonia (1)
M2	A27V	All 4 Bulgarian strains	Albania (1), Macedonia (6)
NS1	N207H	A/Bulgaria/1452/2018; A/Bulgaria/1534/2018	Albania (1), Macedonia (1)

Four Bulgarian viruses (A/Bulgaria/1347/2018, A/Bulgaria/1376/2018, A/Bulgaria/1452/2018 and A/Bulgaria/1534/2018) underwent whole-genome sequencing. Encoded internal protein sequences were compared with those of A/Singapore/INFIMH-16-0019/2016 (EPI_ISL_275709) and other viruses from Albania (EPI_ISL_380070 - EPI_ISL_380074) and Macedonia (EPI_ISL_362159 - EPI_ISL362168).

Analysis of internal proteins in A(H1N1)pdm09 and A(H3N2) viruses revealed the presence of the number of amino acid substitutions, mainly in the PB2 protein, but their biological significance and clinical relevance is unknown.

Antiviral susceptibility testing of influenza viruses circulating in Bulgaria, carried out by means of genotypic and phenotypic methods, showed that all studied type A viruses were susceptible to the within country licensed NA inhibitors oseltamivir and zanamivir. These investigations are necessary to allow rapid detection of viruses with reduced susceptibility or resistance to available antiviral drugs. Global surveillance of influenza virus susceptibility to NA inhibitors has shown very low levels (<1 %) of antiviral resistance among recently circulating influenza viruses [52].

### Conclusion

The 2018/2019 influenza season in Bulgaria was caused by genetically diverse A(H1N1)pdm09 and A(H3N2) viruses that co-circulated. The increased numbers of serious and fatal cases of influenza during the season could be explained by predominance of recently emerged subclade 6B.1A influenza A(H1N1)pdm09 viruses and low vaccination coverage (2–3 % of the population). Continuous monitoring of circulating influenza viruses is necessary in order to evaluate the burden of influenza infection and to adopt an effective national strategy for the control and prevention of influenza.

## Supplementary Data

Supplementary material 1

## References

[1] WHO (2018a). Influenza fact sheet. influenza (seasonal) fact sheet. 6 November 2018; available at. https://www.who.int/news-room/fact-sheets/detail/influenza-(seasonal).

[2] Gerhard W, Yewdell J, Frankel ME, Webster R (1981). Antigenic structure of influenza virus haemagglutinin defined by hybridoma antibodies. Nature.

[3] Wiley DC, Skehel JJ (1987). The structure and function of the hemagglutinin membrane glycoprotein of influenza virus. Annu Rev Biochem.

[4] Wilson IA, Cox NJ (1990). Structural basis of immune recognition of influenza virus hemagglutinin. Annu Rev Immunol.

[5] Koel BF, Burke DF, Bestebroer TM, van der Vliet S, Zondag GCM (2013). Substitutions near the receptor binding site determine major antigenic change during influenza virus evolution. Science.

[6] Koel BF, Mögling R, Chutinimitkul S, Fraaij PL, Burke DF (2015). Identification of amino acid substitutions supporting antigenic change of influenza A(H1N1)pdm09 viruses. J Virol.

[7] Bedford T, Suchard MA, Lemey P, Dudas G, Gregory V (2014). Integrating influenza antigenic dynamics with molecular evolution. Elife.

[8] Harvey WT, Benton DJ, Gregory V, Hall JPJ, Daniels RS (2016). Identification of Low- and High-Impact Hemagglutinin Amino Acid Substitutions That Drive Antigenic Drift of Influenza A(H1N1) Viruses. PLoS Pathog.

[9] Skehel JJ, Stevens DJ, Daniels RS, Douglas AR, Knossow M (1984). A carbohydrate side chain on hemagglutinins of Hong Kong influenza viruses inhibits recognition by a monoclonal antibody. Proc Natl Acad Sci U S A.

[10] Zost SJ, Parkhouse K, Gumina ME, Kim K, Diaz Perez S (2017). Contemporary H3N2 influenza viruses have a glycosylation site that alters binding of antibodies elicited by egg-adapted vaccine strains. Proc Natl Acad Sci U S A.

[11] EU case definitions Available at. https://ecdc.europa.eu/en/infectious-diseases-public-health/surveillance-and-disease-data/eu-case-definitions.

[12] Matrosovich M, Matrosovich T, Carr J, Roberts NA, Klenk H-D (2003). Overexpression of the alpha-2,6-sialyltransferase in MDCK cells increases influenza virus sensitivity to neuraminidase inhibitors. J Virol.

[13] WHO (2011b). Manual for the laboratory diagnosis and virological surveillance of influenza. http://whqlibdoc.who.int/publications/2011/9789241548090_eng.pdf.

[15] GISAID Available at. https://www.gisaid.org/.

[16] RaxML v8.2X Available at. https://cme.h-its.org/exelixis/software.html.

[17] Treesub Available at. https://github.com/tamuri/treesub/blob/master/README.md.

[18] FigTree Available at. http://tree.bio.ed.ac.uk/software/figtree/.

[19] Adobe Illustrator CC (2015). 3. Available at.

[20] MEGA Molecular Evolutionary Genetics Analysis (MEGA, version 6.06) software. Available at. http://www.megasoftware.net/.

[21] FluSurver Available at:. http://flusurver.bii.a-star.edu.sg.

[22] Hussain S, Miller JL, Harvey DJ, Gu Y, Rosenthal PB (2015). Strain-Specific antiviral activity of iminosugars against human influenza A viruses. J Antimicrob Chemother.

[23] Beer K, Dai M, Howell S, Rijal P, Townsend AR (2018). Characterization of neutralizing epitopes in antigenic site B of recently circulating influenza A(H3N2) viruses. J Gen Virol.

[24] WHO (2019c). Worldwide Influenza Centre, London. September 2019 Interim Report. Report prepared for the WHO annual consultation on the composition of influenza vaccine for the Southern Hemisphere 2020. 23rd – 26th September 2019. Available at. https://www.crick.ac.uk/sites/default/files/201910/CrickSH2019VCMreport_v2.pdf.

[25] Caton AJ, Brownlee GG, Yewdell JW, Gerhard W (1982). The antigenic structure of the influenza virus A/PR/8/34 hemagglutinin (H1 subtype). Cell.

[26] Graham M, Liang B, Van Domselaar G, Bastien N, Beaudoin C (2011). Nationwide molecular surveillance of pandemic H1N1 influenza A virus genomes: Canada, 2009. PLoS One.

[27] Maurer-Stroh S, Ma J, Lee RTC, Sirota FL, Eisenhaber F (2009). Mapping the sequence mutations of the 2009 H1N1 influenza A virus neuraminidase relative to drug and antibody binding sites. Biol Direct.

[28] Colman PM, Hoyne PA, Lawrence MC (1993). Sequence and structure alignment of paramyxovirus hemagglutinin-neuraminidase with influenza virus neuraminidase. J Virol.

[29] Belshe RB, Smith MH, Hall CB, Betts R, Hay AJ (1988). Genetic basis of resistance to rimantadine emerging during treatment of influenza virus infection. J Virol.

[30] Fang Q, Gao Y, Chen M, Guo X, Yang X (2014). Molecular epidemiology and evolution of A(H1N1)pdm09 and H3N2 virus during winter 2012-2013 in Beijing, China. Infect Genet Evol.

[31] WHO (2016d). Summary of neuraminidase amino acid substitutions associated with reduced inhibition by neuraminidase inhibitors (Last updated 26 April 2018).. http://www.who.int/influenza/gisrs_laboratory/antiviral_susceptibility/nai_overview/en/.

[32] Takashita E, Daniels RS, Fujisaki S, Gregory V, Gubareva LV (2020). Global update on the susceptibilities of human influenza viruses to neuraminidase inhibitors and the cap-dependent endonuclease inhibitor baloxavir, 2017-2018. Antiviral Res.

[33] Korsun NS, Angelova SG, Trifonova IT, Georgieva IL, Tzotcheva IS (2019). Predominance of influenza B/Yamagata lineage viruses in Bulgaria during the 2017/2018 season. Epidemiol Infect.

[34] Segaloff H, Melidou A, Adlhoch C, Pereyaslov D, Robesyn E (2019). Co-circulation of influenza A(H1N1)pdm09 and influenza A(H3N2) viruses, World Health Organization (WHO) European Region, October 2018 to February 2019. Euro Surveill.

[35] Korsun N, Angelova S, Teodosieva A (2016). Virological surveillance of influenza in four recent Post-Pandemic seasons (2010/11 to 2013/14) in Bulgaria. Cent Eur J Public Health.

[36] Korsun N, Angelova S, Gregory V, Daniels R, Georgieva I (2017). Antigenic and genetic characterization of influenza viruses circulating in Bulgaria during the 2015/2016 season. Infect Genet Evol.

[37] Ilyicheva T, Durymanov A, Susloparov I, Kolosova N, Goncharova N (2016). Fatal cases of seasonal influenza in Russia in 2015-2016. PLoS One.

[38] Horwood PF, Karlsson EA, Horm SV, Ly S, Heng S (2019). Circulation and characterization of seasonal influenza viruses in Cambodia, 2012-2015. Influenza Other Respir Viruses.

[39] Strengell M, Ikonen N, Ziegler T, Julkunen I (2011). Minor changes in the hemagglutinin of influenza A(H1N1)2009 virus alter its antigenic properties. PLoS One.

[40] Gao J, Couzens L, Burke DF, Wan H, Wilson P (2019). Antigenic Drift of the Influenza A(H1N1)pdm09 Virus Neuraminidase Results in Reduced Effectiveness of A/California/7/2009 (H1N1pdm09)-Specific Antibodies. mBio.

[41] WHO (2019e). Recommended composition of influenza virus vaccines for use in the 2019-2020 northern hemisphere influenza season; Available at:. https://www.who.int/influenza/vaccines/virus/recommendations/2019_20_north/en/.

[42] Glinsky GV (2010). Genomic analysis of pandemic (H1N1) 2009 reveals association of increasing disease severity with emergence of novel hemagglutinin mutations. Cell Cycle.

[43] Kolosova NP, Ilyicheva TN, Danilenko AV, Bulanovich JA, Svyatchenko SV (2019). Severe cases of seasonal influenza in Russia in 2017-2018. PLoS One.

[44] Andrés C, Peremiquel-Trillas P, Gimferrer L, Piñana M, Codina MG (2019). Molecular influenza surveillance at a tertiary university hospital during four consecutive seasons (2012-2016) in Catalonia, Spain. Vaccine.

[45] Chutinimitkul S, Herfst S, Steel J, Lowen AC, Ye J (2010). Virulence-associated substitution D222G in the hemagglutinin of 2009 pandemic influenza A(H1N1) virus affects receptor binding. J Virol.

[46] Liu Y, Childs RA, Matrosovich T, Wharton S, Palma AS (2010). Altered receptor specificity and cell tropism of D222G hemagglutinin mutants isolated from fatal cases of pandemic A(H1N1) 2009 influenza virus. J Virol.

[47] Rambaut A, Pybus OG, Nelson MI, Viboud C, Taubenberger JK (2008). The genomic and epidemiological dynamics of human influenza A virus. Nature.

[48] Kawakami C, Yamayoshi S, Akimoto M, Nakamura K, Miura H (2019). Genetic and antigenic characterisation of influenza A(H3N2) viruses isolated in Yokohama during the 2016/17 and 2017/18 influenza seasons. Euro Surveill.

[49] Schulze IT (1997). Effects of glycosylation on the properties and functions of influenza virus hemagglutinin. J Infect Dis.

[50] Simon B, Pichon M, Valette M, Burfin G, Richard M (2019). Whole Genome Sequencing of A(H3N2) Influenza Viruses Reveals Variants Associated with Severity during the 2016–2017 Season. Viruses.

[51] Wedde M, Biere B, Wolff T, Schweiger B (2015). Evolution of the hemagglutinin expressed by human influenza A(H1N1)pdm09 and A(H3N2) viruses circulating between 2008-2009 and 2013-2014 in Germany. Int J Med Microbiol.

[52] Al Khatib HA, Al Thani AA, Gallouzi I, Yassine HM (2019). Epidemiological and genetic characterization of pH1N1 and H3N2 influenza viruses circulated in MENA region during 2009-2017. BMC Infect Dis.

[53] Hammond A, Hunda K, Laurenson-Schafer H, Cozza V, Maharjan B (2019). Review of the 2018–2019 influenza season in the Northern hemisphere. Wkly Epidemiol Rec.

