# Supplementary material for: Genetic diversity of influenza A viruses circulating in Bulgaria during the 2018–2019 winter season
Source: J Med Microbiol. 2020 May 27;69(7):986–98. doi: 10.1099/jmm.0.001198 (PMC7481746; doi:10.1099/jmm.0.001198)
Supplement: Supplementary material 1 [file jmm-69-986-s001.pdf]

Supplementary Table S1. GISAID virus/sequence identification/accession numbers used in this study

A(H1N1)pdm09 strains	Accession number	A(H3N2) strains	Accession number
A/Michigan/45/2015	EPI_ISL_237793	A/Singapore/INFIMH-16-0019/2016	EPI_ISL_344420
A/California/7/2009	EPI_ISL_391380	A/Texas/50/2012	EPI_ISL_132908
A/Bayern/69/2009	EPI_ISL_68758	A/Stockholm/6/2014	EPI_ISL_162144
A/Lviv/N6/2009	EPI_ISL_62012	A/Switzerland/9715293/2013	EPI_ISL_16214
A/Astrakhan/1/2011	EPI_ISL_90787	A/Norway/2620/2018	EPI_ISL_314181
A/Hong Kong/5659/2012	EPI_ISL_127652	A/Hong Kong/681/2018	EPI_ISL_312272
A/Slovenia/2903/2015	EPI_ISL_206099	A/Netherlands/10260/2018	EPI_ISL_309310
A/Paris/1447/2017	EPI_ISL_291688	A/LaRioja/2202/2018	EPI_ISL_31492
A/Switzerland/3330/2017	EPI_ISL_294121	A/Norway/3275/2018	EPI_ISL_332813
A/Norway/3433/2018	EPI_ISL_332840	A/Baden-Wurttemberg/132/2019	EPI_ISL_358021
A/Belgium/G0024/2019	EPI_ISL_355283	A/Greece/1456/19/2019	EPI_ISL_355042
A/Bayern/68/2019	EPI_ISL_355282	A/Grenoble/46/2019	EPI_ISL_355049
A/Greece/1383/19/2019	EPI_ISL_358057	A/Belgium/S0334/2019	EPI_ISL_355023
A/Macedonia/719/2019	EPI_ISL_355391	A/Latvia/02-013062/2019	EPI_ISL_35505
A/Bremen/19/2019	EPI_ISL_358050	A/Bayern/96/2019	EPI_ISL_35802
A/Bucuresti/239839/2019	EPI_ISL_355294	A/Malta/516156/2019	EPI_ISL_347720
A/England/732/2018	EPI_ISL_355317	A/Georgia/917/2018	EPI_ISL_341958
A/Lyon/CHU/R18.134.99/2018	EPI_ISL_355382	A/Israel/10442/2018	EPI_ISL_347150
A/Andalucia/276/2019	EPI_ISL_355265	A/Bucuresti/239696/2018	EPI_ISL_338743
A/Estonia/118397/2018	EPI_ISL_338045	A/Serbia/5270/2019	EPI_ISL_363119
A/Serbia/5237/2019	EPI_ISL_363048	A/Macedonia/723/2019	EPI_ISL_35507
A/Turkey/55/2019	EPI_ISL_355472	A/Albania/9076/2019	EPI_ISL_342127
A/Bosnia and Herzegovina/2760/2019	EPI_ISL_341831	A/Moscow/241/2018	EPI_ISL_34211
A/Parma/182/2018	EPI_ISL_338490	A/Ukraine/8233/2019	EPI_ISL_36481
A/St.Peteresburg/RII-260/2019	EPI_ISL_361901	A/Nordrhein-Westfalen/132/2017	EPI_ISL_29423
A/Switzerland/2656/2017	EPI_ISL_294119	A/Greece/4/2017	EPI_ISL_25737
A/Latvia/03-012374/2019	EPI_ISL_355371	A/Norway/4436/2016	EPI_ISL_239785
A/Greece/1170/19/2019	EPI_ISL_358056	A/Belgium/S0039/2019	EPI_ISL_355017
A/Luxembourg/2575/2019	EPI_ISL_355376	A/Hong Kong/656/2018	EPI_ISL_312267
A/Brandenburg/2/2019	EPI_ISL_355289	A/Switzerland/8060/2017	EPI_ISL_29425
A/Ireland/84630/2018	EPI_ISL_338060	A/Bretagne/1413/2017	EPI_ISL_29237
A/Brisbane/02/2018	EPI_ISL_330190	A/Hong Kong/675/2018	EPI_ISL_31226
A/Cyprus/FA13/2019	EPI_ISL_355302	A/Lyon/2183/2018	EPI_ISL_355064
A/Saint-Etienne/137/2019	EPI_ISL_355447	A/Hong Kong/7295/2014	EPI_ISL_168978
A/Croatia/5327/2018	EPI_ISL_355297	A/Norway/2516/2018	EPI_ISL_314674
A/Madrid/209/2019	EPI_ISL_355392	A/Palermo/334/2018	EPI_ISL_342023
A/Baden-Wurttemberg/7/2019	EPI_ISL_355280	A/Lyon/2296/2018	EPI_ISL_35506
A/Montenegro/146/2019	EPI_ISL_355418	A/Saarland/7/2019	EPI_ISL_355102
A/Bucuresti/239594/2019	EPI_ISL_355293	A/Israel/8701/2018	EPI_ISL_347158
A/Sachsen/28/2019	EPI_ISL_355445	A/Hong Kong/4801/2014	EPI_ISL_16555
A/Malta/53480/2019	EPI_ISL_355397	A/Hong Kong/5738/2014	EPI_ISL_165629
A/Slovenia/888/2019	EPI_ISL_363056	A/Lithuania/MB34980/2018	EPI_ISL_341987
A/Greece/351/2019	EPI_ISL_355346	A/Sachsen-Anhalt/31/2019	EPI_ISL_355103
A/Rheinland-Pfalz/27/2019	EPI_ISL_355442	A/Kansas/14/2017	EPI_ISL_31283
A/Grenoble/43/2019	EPI_ISL_355347	A/England/538/2018	EPI_ISL_31415
A/Nordrhein-Westfalen/90/2019	EPI_ISL_358062	A/Berlin/28/2019	EPI_ISL_355024
A/Berlin/26/2019	EPI_ISL_355286	A/Hessen/42/2019	EPI_ISL_358037
A/Croatia/5331/2018	EPI_ISL_355298	A/Bayern/53/2019	EPI_ISL_355009

A/Malta/53336/2019	EPI_ISL_355394	A/Lyon/95/2019	EPI_ISL_355068
A/Greece/341/2019	EPI_ISL_355345	A/Belgium/S0275/2019	EPI_ISL_355021
A/Georgia/187/2019	EPI_ISL_341854	A/Valladolid/26/2019	EPI_ISL_355127
A/Ukraine/8317/2019	EPI_ISL_364890	A/Israel/10464/2018	EPI_ISL_341967
A/Albania/9168/2019	EPI_ISL_355261		
A/Antalya/5/2018	EPI_ISL_336241		
A/Macedonia/711/2019	EPI_ISL_355388		
A/Andalucia/190267/2018	EPI_ISL_355262		
A/Poland/261/2019	EPI_ISL_338495		
